# Couch modeling optimization for tomotherapy planning and delivery

**DOI:** 10.1002/acm2.12686

**Published:** 2019-07-25

**Authors:** Wataru Okada, Masao Tanooka, Keisuke Sano, Mayuri Shibata, Hiroshi Doi, Masayoshi Miyazaki, Ryuta Nakahara, Masaki Sueoka, Hitomi Suzuki, Masayuki Fujiwara, Taisuke Inomata, Koichiro Yamakado

**Affiliations:** ^1^ Department of Radiology Hyogo College of Medicine Nishinomiya Japan; ^2^ Department of Radiotherapy Takarazuka City Hospital Takarazuka Japan; ^3^ Department of Radiation Oncology Kindai University Faculty of Medicine Sayama Japan

**Keywords:** TomoTherapy, couch modeling, treatment planning system, planning support system

## Abstract

We sought to validate new couch modeling optimization for tomotherapy planning and delivery. We constructed simplified virtual structures just above a default setting couch through a planning support system (MIM Maestro, version 8.2, MIM Software Inc, Cleveland, OH, USA). Based on ionization chamber measurements, we performed interactive optimization and determined the most appropriate physical density of these virtual structures in a treatment planning system (TPS). To validate this couch optimization, Gamma analysis and these statistical analyses between a three‐dimensional diode array QA system (ArcCHECK, Sun Nuclear, Melbourne, FL, USA) results and calculations from ionization chamber measurements were performed at 3%/2 mm criteria with a threshold of 10% in clinical QA plans. Using a virtual model consisting of a center slab density of 4.2 g/cm^3^ and both side slabs density of 1.9 g/cm^3^, we demonstrated close agreement between measured dose and the TPS calculated dose. Agreement was within 1% for all gantry angles at the isocenter and within 2% in off‐axis plans. In validation of the couch modeling in a clinical QA plan, the average gamma passing rate improved approximately 0.6%–5.1%. It was statistically significant (*P* < 0.05) for all treatment sites. We successfully generated an accurate couch model for a TomoTherapy TPS by interactively optimizing the physical density of the couch using a planning support system. This modeling proved to be an efficient way of correcting the dosimetric effects of the treatment couch in tomotherapy planning and delivery.

## 1 INTRODUCTION

Carbon‐fiber flat top couches are widely used for radiotherapy.^1^ These couches have heterogeneous absorption properties when beams pass through the couch before entering the patient.^2^, ^3^, ^4^, ^5^, ^6^, ^7^, ^8^, ^9^, ^10^, ^11^, ^12^, ^13^, ^14^, ^15^, ^16^, ^17^, ^18^ Several authors have reported that the failure to factor in couch attenuation for beams sent in the posteroanterior direction can cause a reduction in target volume coverage.^10^, ^11^, ^12^ The American Association of Physicists in Medicine (AAPM) task group report 176 recommends that the beam intensity attenuation by the couch should be taken into account by the treatment planning system (TPS).^8^ From its earliest version, TomoTherapy (Accuray, Sunnyvale, CA, USA) planning software has implemented this such that this virtual couch has appropriate predefined physical densities and is commissioned sufficiently.

However, Kong et al. reported that for AP treatment in TomoTherapy, with about 50% rear dose contribution, the passing rate of gamma analysis deteriorated to 91.28% (3%/3 mm) even when using a two‐dimensional array ion chamber device with angular dependence correction. They concluded that in pretreatment plan verification, the greater the dose contribution from the rear, the poorer the agreement between the measured dose and TPS.^19^ Similar to their report, we have experienced discrepancies between actual measurement values and planned values in IMRT verification, especially when using the TomoDirect plan, which sends some fixed gantry‐angle beams through the couch in the PA direction.

The dose output stability of the newest generation of TomoTherapy delivery systems was achieved with the addition of a dose servo‐controlled system called DCS. Smilowitz et al. reported that the standard deviation in the monitor chamber daily output varies < 0.5%, making it unlikely that this is the cause of these dose discrepancies.^20^

We suspected that default settings of the couch may be inaccurate. However, it is not currently possible to override the predefined physical density of the couch.

Here, therefore, we developed and validated a new couch modeling optimization for tomotherapy planning and delivery.

## 2 MATERIALS AND METHODS

### 2.1 Ionization chamber measurements for verification of TPS accuracy

To evaluate the accuracy of the couch model in the TomoTherapy’s TPS (Accuray Precision version 1.1.1.1: Accuray, Sunnyvale, CA, USA), we inserted an ionization chamber, Exradin Model A1SL (Standard Imaging, Middleton, WI, USA) into the center of a 15.0 cm ϕ Quasar cylindrical phantom (Modus Medical Devices Inc., North Routledge Park, USA), and scanned with a 16‐slice multidetector CT scanner, Aquilion LB (Canon Medical Systems Co., Tochigi, Japan), with an axial section thickness of 2.0 mm and 700 mm field of view (FOV). Those images were exported to a TPS.

Forward planning (included in TomoDirect) mode with a reference field size of 10 × 5 cm^2^ was used for verification of the couch model in TPS in order to calculate actual couch attenuation. The beam angles used in the model validation were 0° and from 120° to 180° in 5° increments. The prescribed dose was 2 Gy per beam at the isocenter. The calculation grid size, field width, and pitch were 1.36 mm × 1.36 mm × 2.0 mm (equivalent to 1 voxel), 5.0 cm, and 0.500, respectively. The reason that we use a pitch of 0.500 is that the maximum pitch allowed for TomoDirect settings when using a field width of 5.0 cm and optimization is also efficient under that condition. Measurements in all sections were performed with the newest TomoTherapy delivery System (Radixact Version.1.1.0.1: Accuray, Sunnyvale, CA, USA). We defined the attenuation rate by the following equation:

(1) $$\text{Attenuation}\;\text{rate}\;\left(\%\right)=\left(\frac{D_{each\;angle}}{D_{0\;deg}}\right)\times 100.$$

where D is the absorbed dose.

### 2.2 Simplified couch modeling optimization in TPS

The TomoTherapy couch top consists of lower and upper pallets which are composed of carbon fiber on the outside and foam on the inside. A very thin copper foil and optical cable assembly called “flat flex circuits” runs between the two pallets, and is connected to the lateral drive assemblies that control lateral movement. To simplify the modeling of these couch structures in the TPS, we constructed virtual structures just above the original couch using the planning support system (MIM Maestro, version .8.2, MIM Software Inc, Cleveland, OH).

**Figure 1:**
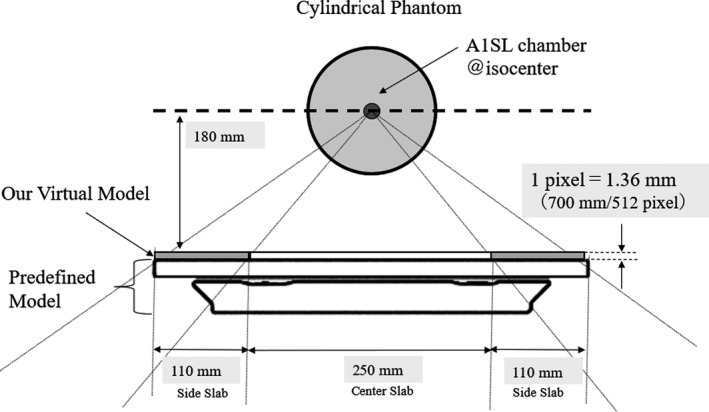
Schematic view of the calculation and measurement geometry. Predefined conventional model and our virtual model (center slab and side slab) are shown

To minimize the geometrical influence of patient and couch, we adopted an ultra‐thin 1‐pixel‐thick slab in the virtual structures. The shape of these structures is shown in Fig. 1. The thick part of the center and thin part of both sides were separated.

Based on the ionization chamber measurements, we performed interactive optimization and determined the most appropriate physical density of these virtual structures in the TPS. We defined the dose difference by the following Eq:

(2) $$\text{Dose}\;\text{Difference}\left(\%\right)=\left(\frac{D_{\mathrm{measured}}-D_{\mathrm{calculated}}}{D_{\mathrm{measured}}}\right)\times 100.$$

where D is the absorbed dose.

### 2.3 Validation of the couch modeling in an off‐axis plan

**Figure 2:**
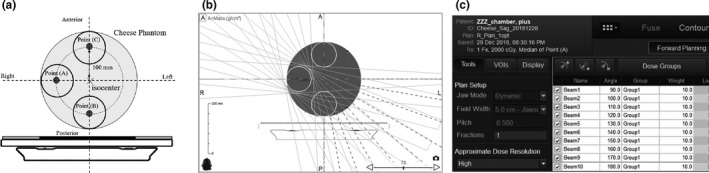
(a) Cylindrical off‐axis targets (100 mm φ × 50 mm) and cheese phantom setup for measurement. The image of the cheese phantom shows three off‐axis target structures. Absolute dose of Point (A)–(C) was measured at the center of each cylindrical target and compared with the treatment planning system calculated dose. (b) Example of the beam arrangement for Point (A) with a 10 × 5 cm field size. (c) Example of the prescription dose and beam weight. These settings made it possible to prescribe exactly 2 Gy per beam

Unlike conventional linacs, movement in the lateral direction of the couch in TomoTherapy is limited to ± 3 cm, and the target is accordingly often located in an off‐axis position. To validate this couch model in a practical off‐axis situation, verification plans were generated for each cylindrical target (10 cm × 5 cm) using a cheese (solid water) phantom for three off‐axis locations [Fig. 2(a) and 2(b)].

As shown in Fig. 2(c), we planned 2 Gy at each beam (20 Gy/10 beams/fraction) to have the same prescribed weight. The calculation grid size, field width, and pitch were similar to those in Section 2.A. The point dose was measured at the center of each cylindrical target (A)–(C) and compared with the TPS calculated dose.

### 2.4 Validation of the couch modeling using clinical QA plans

To validate this couch model optimization, the data of 70 patients who underwent treatment with TomoHelical and TomoDirect plans between September 2018 and December 2018 at our institution were selected. TomoDirect plans with no posteroanterior beam direction were excluded.

**Figure 3:**
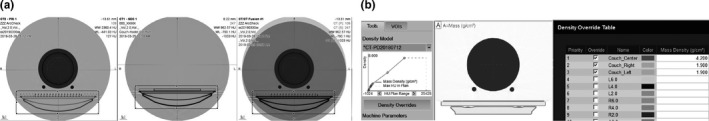
Introduction of the virtual structure mounted on the couch in the delivery quality assurance process. (a) First, an image for the CT couch with virtual structures created in advance and ArcCHECK images need to be opened in the same session in MIM. Next, box‐based image registration needs to be performed in the area of the couch to locate virtual structures immediately above the couch exactly. The ArcCHECK image accompanying the virtual structures should be mounted on the CT couch. (b) After exporting to treatment planning system, an image is registered as a phantom image with accompanying virtual structures and a default setting of couch is inserted. Finally, these structures are overridden with the physical density decided in Section 2.B

Optimized physical densities were adapted in the typical delivery quality assurance (DQA) plan of these patients to evaluate the impact of the treatment couch on the dosimetry validation using a three‐dimensional diode array (ArcCHECK, Sun Nuclear Corporation, Melbourne, FL). Figure 3(a) and 3(b) depict the introduction of the virtual structure mounted on the couch using the planning support system in the DQA process. IGRT using MVCT to minimize couch sag and other positioning errors was performed before all of the ArcCHECK measurements.

Gamma analysis between ArcCHECK results and our calculations was performed at 3% dose difference and 2 mm distance‐to‐agreement criteria with a threshold of 10%. Statistical analyses (paired *t*‐test) were performed using IBM SPSS Statistics software, version 22 (IBM Corp., Armonk, NY, USA).

### 2.5 Surface dose evaluation for couch modeling optimization

According to AAPM Task Group Report 176, it is necessary to confirm both attenuation and surface dose to optimize attenuation property by the couch.^8^ To investigate the validity of couch modeling optimization on surface dose, a 15‐cm thickness water equivalent slab phantom (RW3, PTW Freiburg, Freiburg, Germany) was scanned with a CT scanner, with an axial section thickness of 2.0 mm and 700 mm FOV. The percentage depth dose (PDD) from the AP and PA directions at the central axis with a 10 × 5 cm^2^ field size was calculated in the TPS. Source‐to‐surface distance was set at 85 cm. The calculation grid size, field width, and pitch were similar to those in Section 2.A. PDD from the PA direction was calculated with and without couch modeling optimization.

The surface dose measurements were carried out with GafChromic film (EBT3, International Specialty Product, NJ, USA) using procedures that were planned by the TPS. Measurements were performed at each 1 mm from 0 to 15 mm and 20 mm depths. The exposed films were scanned and digitized (Vidar DosimetryPRO advantage, Vidar System Corp., Herndon, USA), and analyzed using commercial film dosimetry software (RIT complete, Radiological Imaging Technology, Colorado Springs, USA). The measurements were repeated three times to acquire an average value.

## 3 RESULTS

### 3.1 Ion chamber measurements for verification of TPS accuracy

As shown in Table 1, attenuation without couch correction ranged from 0.9% to 9.9%, depending on the beam angles. The greatest attenuation was observed at the gantry angle of 150°, where the beam is passing through the flat flex circuits. The conventional couch modeling narrowed the range to 0.2%–5.4%.

**Table 1:** Modeling of the TomoTherapy couch top with the Precision TPS using different combinations of the couch center slab and both side slabs for a 10 cm × 5 cm field size

Gantry Angle (degrees)	Attenuation rate without couch optimization (%)	Attenuation rate with couch optimization (%)		Dose difference (%) Assigned physical density (g/cm^3^)					
			Side slabs	0	1.7	1.8	1.9	2	2.1
			Center slab	0	4	4.1	4.2	4.3	4.4
0	—			—	—	—	—	—	—
120	0.2	0.9		−0.5	−0.1	0.1	0.1	0.1	0.1
125	0.2	3.4		−1.8	0.5	0.7	0.9	1.3	1.3
130	0.2	3.6		−2.7	−0.3	−0.3	0.0	0.4	0.6
135	4.3	7.4		−3.3	−1.2	−1.2	−1.0	−0.6	−0.4
140	5	9.9		−3.6	0.6	0.8	0.8	1.0	1.2
145	5.4	9.9		−3.3	0.4	0.6	0.6	0.6	0.9
150	5	9.9		−4.2	−0.1	0.1	0.1	0.1	0.3
155	3.6	8.1		−4.1	−0.3	−0.1	−0.1	1.0	1.2
160	3.2	7.4		−4.0	−0.5	−0.3	−0.3	0.6	0.8
165	2.9	7		−4.0	−0.6	−0.4	−0.2	0.6	0.8
170	2.9	6.8		−4.0	−0.9	−0.6	−0.6	0.4	0.6
175	2.7	6.8		−4.0	−0.6	−0.4	−0.4	0.4	0.6
180	2.3	5.9		−3.7	−0.7	−0.5	−0.3	1.2	1.4
			Mean	−3.3	−0.3	−0.1	0.0	0.6	0.7

TPS Prescription Dose (Gy) = 2.0.

### 3.2 Simplified couch modeling optimization in TPS

**Figure 4:**
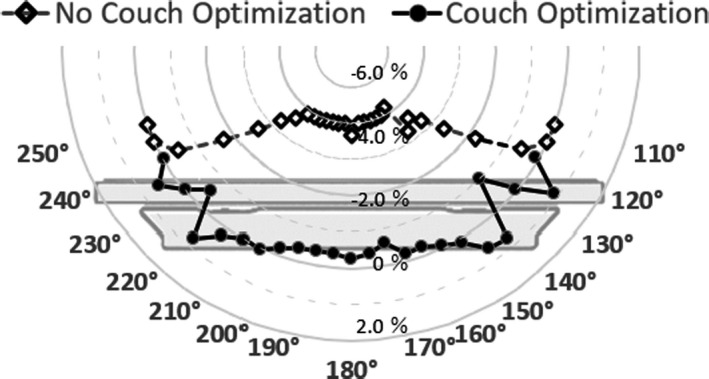
Dose differences with and without couch optimization are shown when the center slab of the virtual structure was assigned a physical density of 4.2 g/cm^2^ and both side slabs were 1.9 g/cm^2^

Modeling of the TomoTherapy couch top in the TPS using different combinations of the center slab and both side slabs is presented in Table 1 for a 10 cm × 5 cm field size. Our modeling decreased the dose difference to < 1.0% when the center slab of the virtual structure was assigned a physical density of 4.2 g/cm^2^ and both side slabs were 1.9 g/cm^2^ (Fig. 4).

### 3.3 Validation of the couch modeling in an off‐axis plan

**Figure 5:**
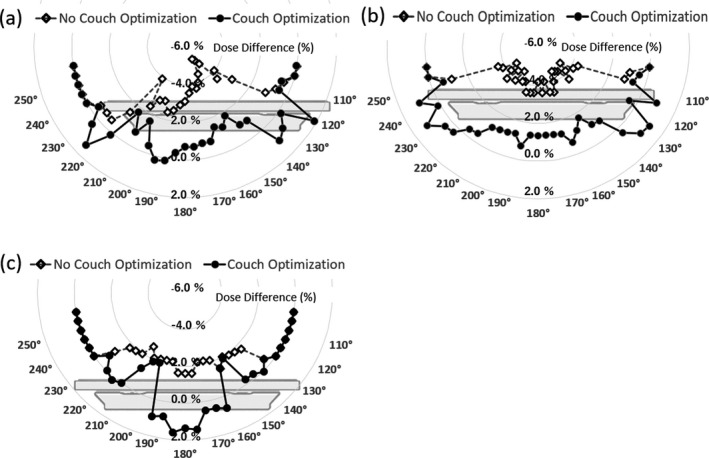
Dose differences with and without couch modeling optimization in an off‐axis plan. (a) Measurement at point (A), (b) measurement at point (B), (c) measurement at point (C)

As shown in Fig. 5(a), 5.8% of the largest discrepancy was observed at a gantry angle of 150° at Point (A), where the path length of the beam through the couch is longest. For all gantry angles, these discrepancies decreased to ≤ 2.0% after using the couch modeling optimization. More fluctuations in dose discrepancy were observed at the interface of these structures and at beam angles that passed through the flat flex circuits compared with isocenter measurements.

### 3.4 Validating the couch modeling using ArcCHECK in clinical QA plans

**Figure 6:**
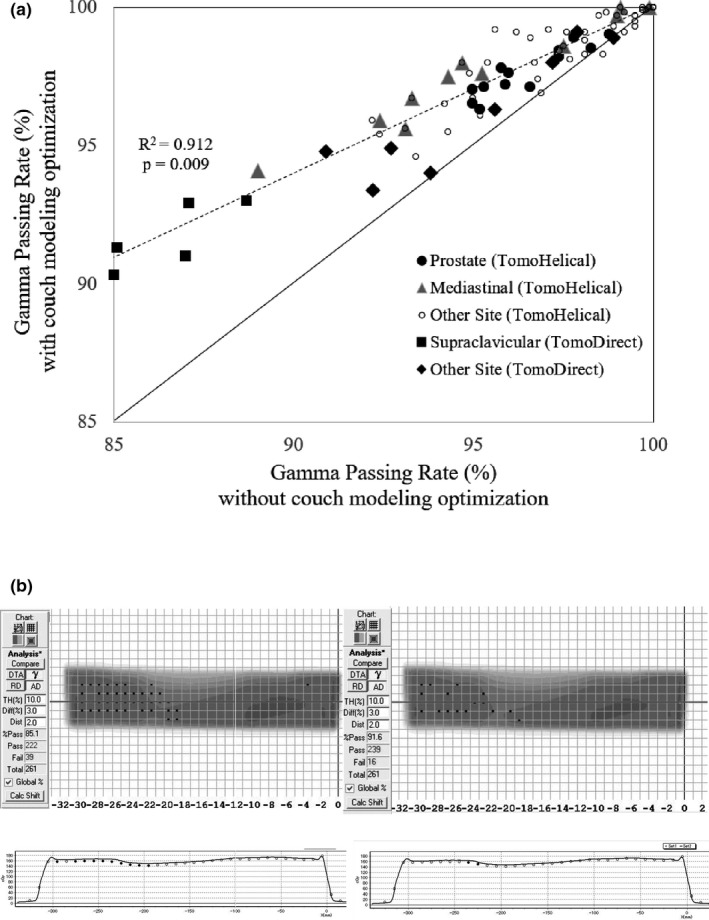
(a) Variation of gamma passing rate with and without couch modeling optimization in each treatment site. (b) One of the results that showed improvement before and after couch optimization in the right supraclavicular area. The underdosed areas were improved by couch modeling optimization

As shown in Table 2, the average gamma passing rate between the measurements and calculations is improved by around 0.6%–5.1%, depending on treatment site, and was statistically significant at all sites. As shown in Fig. 6(a), the lower the gamma passing rate without couch modeling optimization was, the greater improvement was obtained (R^2^ = 0.912 *P* = 0.009). The largest improvement with APPA beams was a supraclavicular plan in a TomoDirect plan of 5.8%. Likewise, the largest improvement with APPA‐modulated beams was a mediastinal TomoHelical plan of 5.1%. Figure 6(b) shows the results before and after couch optimization in the right supraclavicular area. The underdosed areas were improved by couch modeling optimization.

**Table 2:** Mean gamma passing rate with and without couch modeling optimization at each treatment site

Treatment site	Technique	(n = 70)	No couch optimization Mean ± SD	Couch optimization Mean ± SD	*P* value
Prostate	Helical	14	96.5 ± 1.3	97.7 ± 0.9	<0.001
Mediastinal	Helical	9	95.2 ± 3.5	97.6 ± 2.0	0.002
Other sites	Helical	34	97.9 ± 1.4	98.5 ± 0.9	<0.001
Supraclavicular	Direct	5	86.6 ± 1.6	91.7 ± 1.2	<0.001
Other sites	Direct	8	94.9 ± 2.9	96.9 ± 2.2	0.012

### 3.5 Surface dose evaluation for couch modeling

**Figure 7:**
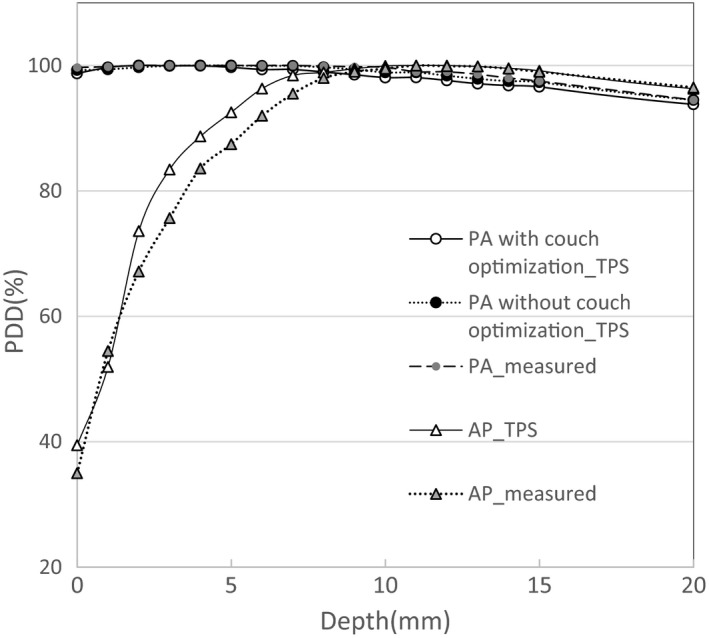
Calculated and measured percentage depth doses (PDDs). Due to passing through the couch, the depth of maximum dose shifted from 11 mm to about 3 mm, and the surface dose increased from about 35%–40% to 98%. Calculated PDD with couch optimization was in good agreement (within 1 mm) with that of the default settings of the couch

As shown in Fig. 7, due to passing through the couch, the depth of maximum dose was shifted from 11 mm to about 3 mm, and the surface dose was increased from about 35%–40% to 98%. The calculated PDD with couch optimization was good agreement (within 1 mm) with that of the default settings of the couch.

## 4 DISCUSSION

The photon beam attenuation patterns of several couch tops have been reported in a number of studies. Njeh et al. reported beam attenuation of 4.9%–10.0% in a 5 × 5 cm^2^ field size for the BrainLAB ICT (BrainLAB, Feldkirchen, Germany).^5^ The 6 MV photon beam measurements of Vanetti et al. with the Varian Exact IGRT (Varian, Palo Alto, CA, USA) couch top (the thinner part) suggested attenuation of 2.3% and 3.1% at gantry angles of 180° and 135°, respectively.^12^ Sedaghatian et al. reported that the maximum attenuation of the Siemens couch (Siemens, Erlangen, Germany) was 5.95% at a 130° gantry angle with 6 MV photon beams.^14^ Smith and colleagues examined the dosimetric properties of the iBEAM evo couch (Medical Intelligence, Schwabmünchen, Germany).^15^ Their ionization chamber measurements showed beam attenuation ranging from 2.7% to a maximum of 4.6% for a 6 MV beam. As shown in Table 1, our results showed similar or slightly greater attenuation than those reported in their study, confirming that the accuracy of the couch modeling by the manufacturer was inaccurate.

We were able to obtain the best agreement between measured and calculated doses with the couch modeling optimization. The level of agreement was < 1.0% at the isocenter, and all dose measurements agreed within ± 2.0% of the TPS calculated dose, which is the generally accepted tolerance of 2%/2 mm of TPS suggested by Venselaar et al.^21^ This confirms that the combination of the couch structure and assigned physical density adopted in this study are reasonable for correcting the couch effects using the TPS.

Using the results of the ArcCHECK‐generated patient QA plan, we found that the influence of the type of couch was greater with the plans having fewer beam ports, such as for supraclavicular nodal irradiation, or with intense AP/PA beams such as that for mediastinal irradiation for the purpose of avoiding normal lung. These results support the fact that the couch greatly affects the dose verification. The few TomoHelical plans in which deterioration of the gamma passing rate was observed were a case in which the dose difference shifted to the plus side due to accuracy of the TPS so that it overcorrected the couch attenuation. Westerly et al. reported that the impact of leaf‐timing inaccuracies on plans with small mean leaf open times (LOTs) can be considerable. Their study suggests reducing this effect and improving delivery efficiency by increasing the pitch.^22^ This may result in further improvement of our DQA results.

According to the result of Section 3.E, the difference of surface dose with and without couch modeling optimization was negligible. Therefore, introducing this method to areas closer to couch is feasible.

A limitation of this study is that the model cannot accurately reproduce the geometric relationship between the couch and target. Therefore, fluctuations in dose discrepancy were observed, especially at the interface of these structures. In addition, a relatively large dose fluctuation was found in beam angles that passed through the flat flex circuits. This might have been due to the failure to factor in the composition of the copper foil and optical cable assembly in the dose calculation. Nevertheless, it is < 2.0%, and therefore at an acceptable level.

In addition, it is necessary to be mindful of dose calculation grid size, as the traditional TomoTherapy TPS planning station downsamples the CT image size from 512 × 512 to 256 × 256 even when using the finest grid size, so the optimal physical density may slightly differ from our report. Therefore, it is recommended that facilities independently investigate this.

Indexed patient immobilization systems are now commonly used to establish reproducible patient positions relative to the couch, and employing such devices provides the best opportunity to accurately account for the couch top during the planning process. To ensure accurate measurement results for off‐axis situations such as that mentioned in Section 2.C, it is necessary to use a robust immobilization system.

TomoTherapy machines come precommissioned to the site from the manufacturer. Upon installation, the user performs a series of acceptance tests to verify that the machine performance is within the specifications. Then, the beam scan data are measured with a 2D water scanning system to verify that they match those measured by the manufacturer at their plant. Finally, IMRT plans are generated, measured with phantoms, and compared for acceptable delivery.^23^

Until now, these acceptance tests and the commissioning test did not include couch modeling validation. Therefore, extra testing by the user is essential for safe delivery of tomotherapy to patients. This report is the first study to carry out couch modeling optimization for tomotherapy. The use of the planning support system made it easy to implement the virtual structure stably to the conventional couch. Our method in this study is very simple, and can be implemented easily at any site.

## 5 CONCLUSION

We generated an accurate couch model for the TomoTherapy planning system by interactively optimizing the physical density of the couch using a planning support system. This modeling proved to be an efficient way of correcting the attenuation effects of the treatment couch in tomotherapy planning and delivery.

## CONFLICT OF INTEREST

The authors have no conflicts of interest to disclose.
